# Glucose Metabolism Changes in Patients with Chronic Hepatitis C Treated with Direct Acting Antivirals

**DOI:** 10.1155/2018/6095097

**Published:** 2018-10-03

**Authors:** Sylvia Drazilova, Martin Janicko, Lubomir Skladany, Pavol Kristian, Marian Oltman, Maria Szantova, Dusan Krkoska, Eva Mazuchova, Lubica Piesecka, Veronika Vahalova, Marek Rac, Ivan Schreter, Ladislav Virag, Tomas Koller, Adriana Liptakova, Miriam Ondrasova, Peter Jarcuska

**Affiliations:** ^1^Department of Internal Medicine, Hospital Poprad, Banícka 803/28, Poprad, 05801, Slovakia; ^2^1st Department of Internal Medicine, PJ Safarik University, Faculty of Medicine and L Pasteur University Hospital, Trieda SNP 1, Kosice, 04001, Slovakia; ^3^2nd Department of Internal Medicine, FD Roosevelt University Hospital, Ludvíka Svobodu 1, Banska Bystrica, 97517, Slovakia; ^4^Department of Infectious Diseases and Travel Medicine, PJ Safarik Unversity, Faculty of Medicine and L Pasteur University Hospital, Rastislavova 43, Kosice, 04001, Slovakia; ^5^Center for Gastroenterology and Hepatology Thalion, Tomášikova 50/C, Bratislava, 83104, Slovakia; ^6^3rd Department of Internal Medicine, Commenius University, Faculty of Medicine and University Hospital, Limbová 5, Bratislava, 83305, Slovakia; ^7^Department of Infectious Diseases and Travel Medicine, Commenius University, Jesenius Faculty of Medicine and University Hospital, Štefanovičova 689/3, Martin, 03601, Slovakia; ^8^Department of Infectious Diseases, Teaching Hospital Nitra, Špitálska 6, Nitra, 95001, Slovakia; ^9^Department of Internal Medicine, Teaching Hospital Nitra, Špitálska 6, Nitra, 95001, Slovakia; ^10^5th Department of Internal Medicine, Commenius University, Faculty of Medicine and University Hospital, Ružinovská 6, Bratislava, 82606, Slovakia; ^11^Department of Microbiology, Commenius University, Faculty of Medicine and University Hospital, Špitálska 13, Bratislava, 81372, Slovakia; ^12^Saint Elisabeth University of Health and Social Sciences, Palackého 1, Bratislava, 81000, Slovakia

## Abstract

**Background and Aims:**

Chronic hepatitis C is a systemic disease and type 2 diabetes mellitus (T2DM) belongs to more common extrahepatic. The aim of this study was to (i) explore the prevalence of impaired fasting glucose (IFG) and T2DM in patients with chronic hepatitis C, (ii) explore the effect of direct acting antivirals (DAA) treatment on the glycemia, and (iii) explore the factors that modulate the effect of DAA treatment on glycemia in patients with chronic hepatitis C.

**Methods:**

We performed a longitudinal retrospective observational study focused on the patients undergoing DAA treatment of chronic hepatitis C. Data about glycemia, history of diabetes, hepatitis C virus, treatment, and liver status, including elastography, were obtained at baseline (before treatment start), at the end of treatment and 12 weeks after the end of treatment. Patients were treated with various regimens of direct acting antivirals.

**Results:**

We included 370 patients; 45.9% had F4 fibrosis. At baseline, the prevalence of T2DM increased with the degree of fibrosis (F0-F2 14.4%, F3 21.3%, and F4 31.8%, p=0.004). Fasting glycemia also increased with the degree of fibrosis (F0-F2 5.75±0.18 F3 5.84±0.17, and F4 6.69±0.2 mmol/L, p=0.001). We saw significant decrease of glycemia after treatment in all patients, but patients without T2DM or IFG from 6.21±0.12 to 6.08±0.15 mmol/L (p=0.002). The decrease was also visible in treatment experienced patients and patients with Child-Pugh A cirrhosis.

**Conclusion:**

We confirmed that the prevalence of either T2DM or IFG increases in chronic hepatitis C patients with the degree of fibrosis. The predictive factors for T2DM were, besides F4, fibrosis also higher age and BMI. Significant decrease of fasting glycemia after the DAA treatment was observed in the whole cohort and in subgroups of patients with T2DM, IFG, cirrhotic, and treatment experienced patients.

## 1. Introduction

About 170 million people were infected with Hepatitis C virus (HCV) in 2013. Overall prevalence is slightly decreasing, mainly due to effective treatment [1]. Active viral replication of HCV is present in about 70 million people worldwide [2]. Chronic hepatitis C may progress to liver cirrhosis. Hepatocellular cancer (HCC) usually occurs in bridging fibrosis (Metavir F3) and cirrhosis (Metavir F4). Decompensated liver cirrhosis and hepatocellular cancer are two most common causes of death of patient with chronic hepatitis C [3].

The treatment by direct acting antivirals (DAA), which inhibit NS3/4a protease and NS5A and NS5B polymerase, leads to sustained virological response (SVR) in almost all infected patients [4]. Achievement of SVR decreases liver-related as well as all-cause mortality in these patients [5].

Chronic hepatitis C is a systemic disease, because it damages also other organs besides liver. Almost three quarters of patients with chronic hepatitis C have extrahepatic manifestations. These may develop well before the diagnosis of chronic hepatitis C [6]. Type 2 diabetes mellitus (T2DM) belongs to more common extrahepatic manifestations of chronic hepatitis C [7]. Insulin resistance is significantly more common in patients with chronic hepatitis C, even with low degree fibrosis, compared to healthy controls. Insulin resistance is also associated with fibrosis progression and portal inflammation [8]. T2DM is significantly more common in patients with HCV related cirrhosis compared to noncirrhotic patients and in chronic hepatitis C patients who failed interferon-based treatment [9]. T2DM is also associated with more frequent occurrence of HCC in patients with chronic hepatitis C [10]. On the other hand, prevalence of HCV infection is higher among T2DM patients compared to nondiabetic patients [11].

About 347 million people world-wide are diagnosed with T2DM [1] and the prevalence is increasing. Fifty-six million people suffer from T2DM in Europe only and estimated prevalence is 8.5% [12]. T2DM is associated with lower life expectancy based on the age of diagnosis [13].

The aim of this study was to

1. explore the prevalence of Impaired fasting glucose (IFG) and T2DM in patients with chronic hepatitis C and various degrees of liver fibrosis
2. explore the effect of DAA treatment on the glycemia levels in patients with chronic hepatitis C
3. explore the factors that modulate the effect of DAA treatment on glycemia in patients with chronic hepatitis C

## 2. Patients and Methods

We designed a retrospective longitudinal observational study focused on the patients undergoing DAA treatment of chronic hepatitis C in multiple centres in Slovakia.

Ethics committee of Poprad Hospital, Banícka 803/28, 05845 Poprad, Slovakia, approved the biomedical research protocol (no. 14/5/2018). Only retrospective anonymized patient data were used; patients signed general informed consent for usage of these data outside standard clinical care. The study was performed in compliance with the Declaration of Helsinki.

### 2.1. Patients

Study included consecutive patients treated for chronic hepatitis C in multiple centres in Slovakia. Exclusion criteria were age less than 18 years, noncompliance, and malignancy with the exception of hepatocellular cancer and localised malignancies of the skin. All patients were treated with standard of care DAA treatment according to guidelines valid at the time of the treatment. DAA treatment regimens included (i) ombitasvir, paritaprevir, ritonavir, and dasabuvir (3D combo), (ii) sofosbuvir and ledipasvir, (iii) grazoprevir and elbasvir, (iv) sofosbuvir monotherapy, (v) sofosbuvir and daclatasvir, (vi) sofosbuvir and velpatasvir, and (vii) sofosbuvir and simeprevir.

### 2.2. Measures

Multiple variables were obtained from patients' documentation retrospectively. Variables included (i) demographics, age and gender; (ii) the parameters of glucose metabolism, fasting plasma glucose, set diagnose of T2DM or IFG, and respective treatment; (iii) information about HCV infection, duration, previous treatment, genotype, serum levels of HCV RNA, DAA treatment and duration, and ribavirin; (iv) presence of coinfections, HBs antigen, and antiHIV antibodies; (v) dyslipidemia and arterial hypertension treatment information; (vi) liver fibrosis and function, fibrosis stage determined by transient elastography expressed in kPa, Child-Pugh score in cirrhotics, presence of HCC, and extrahepatic manifestations of HCV infection; (vii) selected results of laboratory tests, total cholesterol, LDL-cholesterol, triglycerides, HDL-cholesterol, creatinine, C-reactive protein, hemoglobin level, neutrophils, platelets, alpha feto-protein, albumin, INR, AST, ALT, ALP, GMT relative to the ULN, and bilirubin both total and conjugated; and (viii) available variables used to calculate noninvasive fibrosis scores APRI [14], FORNS [15], and FIB-4 [16].

All laboratory tests and virus-related tests were done by standardized, routinely used laboratory methods. Each participating centres performed these tests independently.

The degree of fibrosis was evaluated either histologically or by transient elastography using FibroScan touch 502 device (Echosens, France). Cut-off values used were 9.5 kPa for F3 (Metavir) stage and 12.5 kPa for F4 (Metavir) fibrosis [17]. Decompensation of liver fibrosis was evaluated by calculation of Child-Pugh score, where B or C class was considered a decompensation.

Data were collected at baseline (before treatment start), at the end of treatment (EoT), and 12 weeks after the end of treatment (EoT12w).

Atherogenic index of plasma was calculated as* log (triglycerides/HDL-cholesterol) *[18]

Impaired fasting glucose was defined as glycemia after overnight fasting from 5.6 mmol/L (100 mg/dL) to 6.9 mmol/L (125 mg/dL) and no T2DM history. Type 2 diabetes mellitus was defined as fasting glycemia ≥7.0mmol/L (≥126 mg/dL) or a history of T2DM or antidiabetic treatment [19].

### 2.3. Statistical Analysis

Data is presented as mean ± standard error of mean or absolute (relative) counts. HCV RNA was analysed after log transformation. First, we compared variables among three categories of fibrosis (Metavir F0-F2 versus F3 versus F4) by using either ANOVA or its nonparametric alternative, Kruskal Wallis for interval and chi-square for categorical variables. Next, we assessed the correlation between fasting glycemia and fibrosis levels (both elastography and noninvasive scores) by Pearson correlation. We also compared baseline fasting glucose and the prevalence of IFG and T2DM between Child-Pugh A and Child-Pugh B/C patients by student* t*-test and chi-squared test, similarly for the comparison between treatment naïve and experienced patients in general and in selected F4 patients. Then we assessed the risk factors for T2DM before treatment by univariate logistic regression. Next, we explored the evolution of glycemia during the DAA treatment (glycemia levels at baseline, EoT, and EoT12w) in the whole cohort and then split by the fibrosis levels, Child-Pugh categories, and treatment experience by Friedman test for three related samples. Finally, we explored the factors that may contribute to the observed decrease of glycemia after treatment by univariate logistic regression.

## 3. Results

Study cohort consisted of 370 patients altogether; there were several variables with missing data. These patients were omitted from analysis on a case-per-case basis.

Only one patient (0.3%) had F0 fibrosis, 51 patients (13.8%) had F1 fibrosis, 66 patients (17.8) had F2 fibrosis, 80 patients (21.6%) had F3 fibrosis, and 170 patients (45.9%) had F4 fibrosis/cirrhosis. There was statistically significant overrepresentation of F4 patients (p<0.0001). Two patients had missing data on the degree of fibrosis. There was no difference in the proportion of treatment experienced patients between Child-Pugh A versus B/C class (119, 73.5% versus 13, 76.5%; p=0,788).

### 3.1. Baseline Associations with Glucose Metabolism Disturbances

Baseline, pretreatment parameters are summarized in Tables 1 and 2.

Patients with more advanced fibrosis were older, had higher BMI, and were more commonly treated for dyslipidemia and arterial hypertension. Extrahepatic manifestations and treatment naïve patients occurred mostly in F0-F2 category.

As seen in Table 2, most patients were treated with 3D combo with ribavirin. There was no significant difference in treatment duration between fibrosis categories. Sustained virological response rate was very high. The differences in routine laboratory parameters between fibrosis groups were in line with expectations, except for creatinine, which was the highest in F0-F2 group. Mean fasting glycemia levels were significantly raising with each category of fibrosis. Patients with F4 fibrosis had significantly lower levels of total and LDL cholesterol.

Out of all F4 patients, two patients (1.2%) were classified into Child-Pugh C class, 15 (8.8%) were classified into Child-Pugh B class, and 153 (90%) were in the Child-Pugh A class. Twenty patients (5.4%) had terminal kidney failure (K/DOQI 5) and were on renal replacement therapy. The majority (78.9%) were F0-F2 fibrosis, 10.5% were F3, and another 10.5% were F4 fibrosis (p<0.0001)

**Figure 1:**
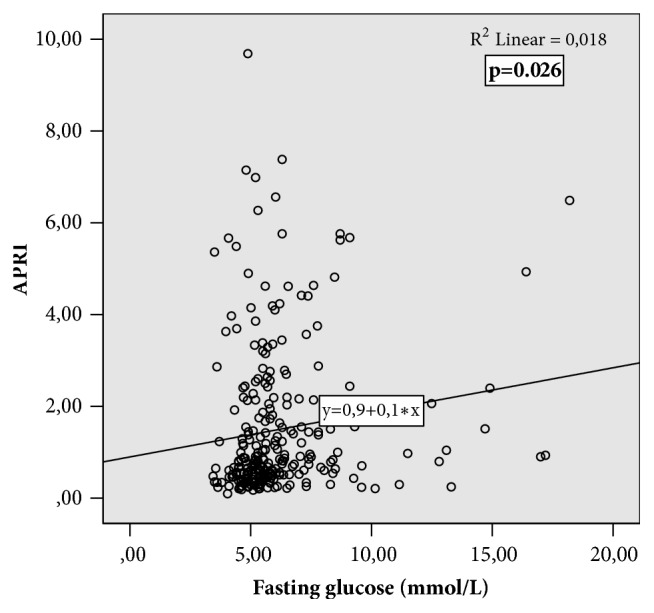
Correlation between fasting glycemia and APRI noninvasive score of fibrosis.

Weak correlations were observed between fasting glycemia and APRI (R^2^ =0.018, p=0.026 (Figure 1)), Forns (R^2^=0.04, p=0.001), and FIB-4 scores (R^2^ = 0.017. p=0.031). Correlations between fasting glycemia and stiffness by transient elastography (R^2^ =0.013, p=0.058) and thrombocytes (R^2^ =-0.009, p=0.071) were also almost significant.

The prevalence of IFG or T2DM significantly increased with the degree of liver fibrosis. In F4 Metavir stage, more than half of the patients had IFG or T2DM. Type 2 DM only was present in 14.4% patients with F0-F2 fibrosis, 21.3% patients with F3, and 31.8% with F4 fibrosis. More than half of the patients had either IFG or T2DM in F4 fibrosis category (Table 3). However, the levels of fasting glycemia or prevalence of IFG or T2DM were not different between Child-Pugh A and B/C patients.

Patients with treatment experience had overall higher levels of fasting glycemia and higher prevalence of IFG but not T2DM. This was not the case in the subgroup of F4 fibrosis patients (Table 4).

Table 5 summarizes a series of univariate logistic regression models that were used to assess the predictors of T2DM in chronic hepatitis C. Only age, BMI, and F4 fibrosis were associated with higher risk of T2DM, even after adjustment for each other. Overall multivariate model containing age, BMI, and the presence of F4 fibrosis had a model fit of R^2^=0.124.

### 3.2. Evolution of Glycemia with DAA Treatment

**Figure 2:**
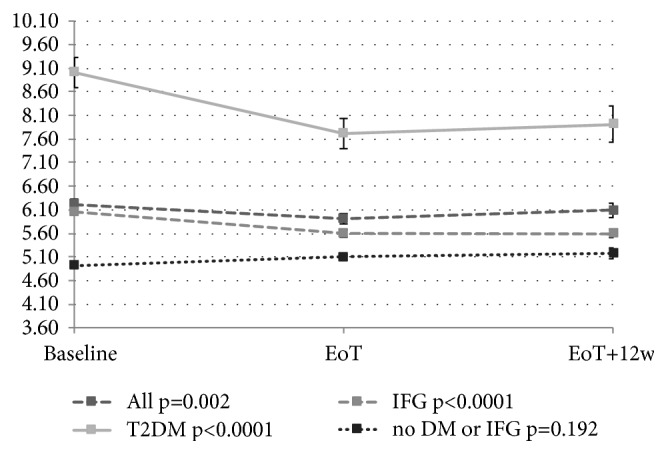
Changes of fasting glycemia with treatment in all patients and subgroups of patients with type 2 diabetes mellitus or impaired fasting glucose. EoT: end of treatment; EoT+12w: 12 weeks after the end of treatment.

**Figure 3:**
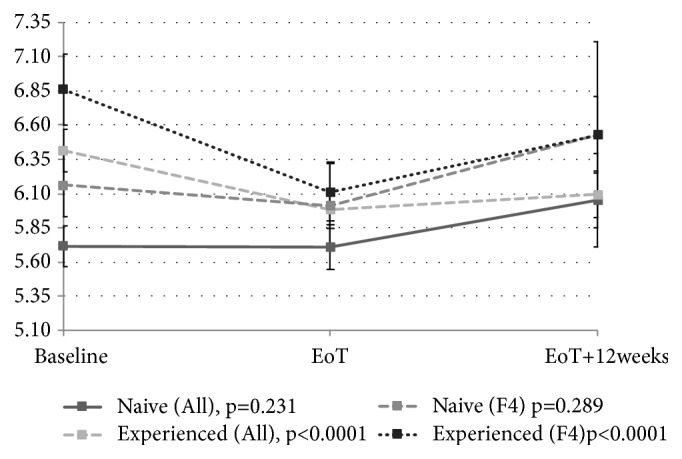
Changes of fasting glycemia with treatment in naive and experienced patients in the whole group and separately for F4 fibrosis patients. EoT: end of treatment; EoT+12w: 12 weeks after the end of treatment.

**Figure 4:**
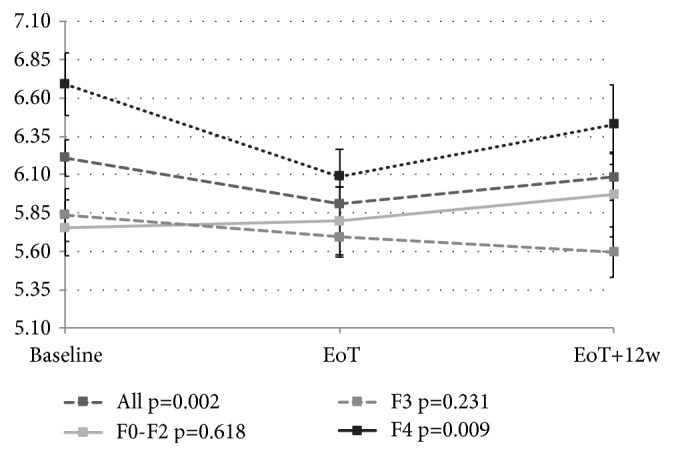
Changes of fasting glycemia with treatment in the whole group and separately for F0-F2, F3, and F4 (Metavir) fibrosis patients. EoT: end of treatment; EoT+12w: 12 weeks after the end of treatment.

**Figure 5:**
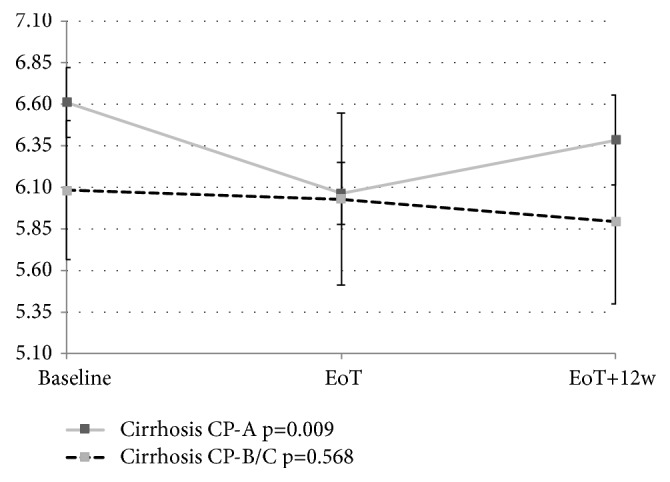
Changes of fasting glycemia with treatment in patients with Child-Pugh A and Child-Pugh B/C cirrhosis. EoT: end of treatment; EoT+12w: 12 weeks after the end of treatment.

Figures 2, 3, 4, and 5 depict the changes of glycemia in all patients and selected subgroups. In Figure 2 a significant decrease of glycemia, in all but patients without T2DM or IFG, is visible.  Figure 3 shows significant decrease of fasting glycemia only in treatment experienced patients. Ultimately, Figure 4 shows that in F4 patients the decrease of fasting glycemia happened only in Child-Pugh A patients.

Thirty T2DM patients were on antidiabetic medication before the start of DAA therapy, 13 were taking oral antidiabetics, and 17 were taking insulin. None of the patients treated with oral antidiabetics had a reduction in the dosage of antidiabetics during the DAA treatment or in 12-week follow-up. Three insulin treated patients needed an insulin dose reduction due to documented hypoglycemia (10% of all treated patients and 17.6% of insulin treated patients).

Finally, we were interested in the predictors of significant (>5%) decrease of glycemia after treatment, which happened in 47.8% of patients. Serial univariate logistic regression showed that only female sex and baseline glycemia predicted the achievement of significant glycemia decrease in the whole cohort. Degrees of fibrosis or treatment experience were not predictors of posttreatment glycemia decrease (Table 6). Baseline glycemia was a significant predictor of fasting glycemia decrease after treatment event after adjustment for age and sex (OR 1.498; 95% CI 1.210-1.854).

## 4. Discussion

This study analysed retrospective data of Slovak patients before the start of DAA treatment for hepatitis C.

Baseline fasting glycemia was increasing with the degree of liver fibrosis. We observed weak correlation between fasting glycemia and APRI score (Figure 1), FIB-4, and Forns index; however, the correlation between fasting glycemia and liver stiffness by transient elastography did not reach statistical significance, probably due to the small sample size. These conflicting results are probably due to low number of analysed patients.

Our results corroborate previously published data that show high prevalence of T2DM in patients with hepatitis C, with prevalence increasing with the degree of fibrosis [9, 11]. Patients with cirrhosis due to hepatitis C have significantly higher prevalence of T2DM compared to different etiologies of cirrhosis [11]

Treatment experienced patients had significantly higher baseline glycemia, higher prevalence of IFG, and higher prevalence of either IFG or T2DM compared to treatment naive patients. It has been shown that insulin resistance is associated with worse outcome of interferon-based treatment [20] and that insulin resistance is associated with higher degree of fibrosis [8]. The highest number of treatment experienced patients was among cirrhotics also in our cohort. Therefore, we expected also higher proportion of T2DM patients in treatment experienced group. When we considered only F4 patients there was no difference between the prevalence of T2DM or IFG between experienced and naive patients or patients with compensated and decompensated cirrhosis.

In our study, only predictive factors for baseline T2DM were higher age, higher BMI, and F4 fibrosis. No association was found between HCV RNA levels, the duration of HCV infection, gender or previous treatment, and T2DM. There is a discrepancy between these results and published data, where the risk of T2DM was associated also with the duration of HCV infection and the response to previous treatment, together with family history of T2DM and insulin sensitivity [1].

Treatment was, as expected, highly effective, with more than 95% achieving sustained virological response, even in F4 fibrosis or Child-Pugh B/C cirrhosis groups. The achievement of SVR may not be the only benefit of DAA treatment. The eradication of the HCV has positive effect also on the extrahepatic manifestations of chronic HCV infection. Three studies have already shown that the achievement of SVR is associated with the decreased risk of future T2DM [9, 21, 22]. In a retrospective study Arase et al. reported that chronic HCV patients that do not respond to interferon-based therapy have almost threefold risk of future T2DM even after adjustment for age, cirrhosis, and prediabetes before the start of the treatment [9]. The achievement of SVR reduced the risk of future T2DM more than twofold in a Spanish prospective study [21]. Similar results were reported in a retrospective analysis from Barcelona [22]. This decrease of T2DM risk was not described in an Italian study with more than 8-year follow-up; however, this study included only small number of patients [23].

### 4.1. Treatment Effect

In this study we also evaluated the dynamics of glycemia before treatment, at EoT, and 12 weeks after EoT in general and in subgroups based on the degree of fibrosis, compensation of cirrhosis, and treatment experience.

Antiviral treatment of chronic hepatitis C may lead to the improvement of glucose metabolism mainly in patients who achieved SVR. During 48-week interferon-based treatment a significant decrease of fasting glucose and glycated hemoglobin (HbA1c) was observed in patients who achieved SVR but not in relapsers [24]. Our study evaluated the changes in fasting glycemia after the EoT and 12 weeks after EoT. We observed significant decrease of fasting glycemia in all patients (p=0.002). This decrease was the highest in diabetics and patients with IFG (p<0.0001 for both). However, glycemia did not change in patients without IFG or T2DM. These results confirm the findings of post hoc analysis of 6 registration phase 3a studies for paritaprevir/ritonavir + dasabuvir + ombitasvir. Treated patients had significant decrease of fasting glycemia compared to placebo patients. This decrease was observed particularly in patients with T2DM and patients with prediabetes. Patients without prediabetes or T2DM experienced a slight increase of fasting glycemia [25].

More studies documented the decrease of fasting glucose levels and HbA1c [26–29], fasting glucose [30], or HbA1c [31, 32] in patients with chronic hepatitis C and T2DM either during the treatment period or at the time of SVR. Significant decrease of fasting glucose occurs in the first four weeks of DAA treatment [25, 30] and persists after the treatment ending. Significant decrease of HbA1c was also observed in patients with chronic hepatitis C treated by DAA after liver transplantation [33]. Only one prospective cohort study failed to show a significant decrease of fasting glycemia nor HbA1c in patients with and without HIV coinfection regardless of the presence of T2DM [34]. Glycemia improvement is not observed after DAA treatment in all patients with chronic hepatitis C. Italian authors documented the decrease of fasting glycemia in 67% and HbA1c in 80% of patients treated with DAA [26]. Egyptian authors observed the improvement of glycemic control in 77.2% of genotype 4 chronic hepatitis C and T2DM patients who achieved SVR twelve weeks after the treatment conclusion [27].

One of the side effects of DAA treatment of chronic hepatitis C in T2DM patient may be hypoglycemia. Spanish authors described a case of symptomatic hypoglycemia in T2DM hepatitis C patient at 18th day of sofosbuvir/ledipasvir treatment despite radical decrease of insulin dosage [35]. Indeed, the reduction of antidiabetic treatment is required in 8%-40% of these patients, particularly in those treated with insulin [26, 27, 29, 31–33]. In our cohort of patient, the reduction of antidiabetic medication occurred in 10% of patients, all of them treated with insulin.

We observed significant decrease of glycemia in F4 fibrosis patients, but not in F3 or F0-F2 fibrosis patients. This is probably due to higher prevalence of T2DM or IFG in F4 fibrosis patients. Moreover, significant decrease of glycemia was observed only in Child-Pugh A, but not B or C cirrhotic patients. Similarly, in the Egyptian study the glycemic control was improved more commonly in Child-Pugh A patients. Since we did not observe any difference between Child-Pugh A and B/C patients in baseline glycemia, prevalence of T2DM or IFG, or treatment experience we suspect it is related to lower liver glycogen stores in decompensated cirrhosis.

When we considered treatment experience, we observed that significant decrease of glycemia occurred only in treatment experienced patients compared to treatment naive patients. Treatment experienced patient had significantly higher baseline fasting glycemia, significantly higher prevalence of IFG or T2DM, and significantly higher rate of F4 fibrosis. Similarly, significant decrease of glycemia occurred only in treatment experienced cirrhotics compared to naive.

Because of very few patients who sustained treatment failure, we could not compare the dynamics of glycemia between patients who did and did not achieve a SVR. In a study from US, patients without cirrhosis who achieved SVR had significantly higher reduction of HbA1c compared to patients who failed the treatment [31].

Insulin resistance and T2DM play the principal role in the pathophysiology of atherosclerosis. Patients with chronic hepatitis C have elevated cardiovascular risk. The achievement of SVR in nondiabetic patients may reduce future T2DM prevalence in this cohort. The achievement of SVR in diabetic patients is associated with improved glycemic control. Thus one could logically extrapolate that the eradication of HCV infection through DAA treatment may indeed lead to the decrease of cardiovascular risk [36].

### 4.2. Limitations

This study has several limitations; probably the greatest is its retrospective design and thus the omission of more important data, e.g., the data on HbA1c before, during, and after DAA treatment. Another limitation is a relatively lower number of participants, out of which only minority had T2DM or IFG. The data is limited also by short time of follow-up after the finish of DAA treatment. Finally, patients received different DAA medications. A prospective multicentric study with larger cohort of patients may be able to explain the changes in the insulin resistance in hepatitis C patient more thoroughly. Such study will be very difficult to undertake, since large proportion of treatment experienced and cirrhotic hepatitis C patients in Europe underwent successful treatment already [37].

## 5. Conclusions

This retrospective study confirmed that the prevalence of either T2DM or IFG increases in chronic hepatitis C patients with the degree of fibrosis; patients with F4 fibrosis had 27.1% prevalence of IFG and 31.8% of T2DM. The predictive factors for T2DM had besides F4 fibrosis also higher age and BMI. Significant decrease of fasting glycemia at the end of treatment and 12 weeks after that was observed in the whole cohort and in subgroups of patients with type 2 diabetes mellitus, impaired fasting glucose, Child-Pugh A cirrhotic patients, treatment experienced patients, and treatment experienced cirrhotics. Long term follow-up may further show if the achievement of SVR after DAA treatment will reduce the risk of future T2DM development similarly to SVR after interferon treatment and if the improvement of glycemic control in patients with T2DM decreases the risk of chronic complications and improves survival.

## Figures and Tables

**Table 1:** Baseline parameters of the study cohort by degrees of fibrosis.

	F0-F2 (Metavir) N=118 Absolute (relative) counts or mean ± SEM	F3 (Metavir) N=80 Absolute (relative) counts or mean ± SEM	F4 (Metavir) N=170 Absolute (relative) counts or mean ± SEM	P
Male sex	58 (49.2)	34 (42.5)	75 (42.4)	0.477

Age (years)	53.64±1.339	60.56±1.297	61.08±0.743	<0.0001

Duration of HCV infection (years)	9.37±0.72	10.82±0.75	11.06±0.59	0.158

BMI	26.17±0.50	27.96±0.51	26.94±0.35	0.037

genotype 1b	93 (78.8)	73 (91.3)	149 (87.6)	0.002
genotype 1a	14 (11.9)	5 (6.3)	4 (2.4)	
genotype 1 (unspecified)	4 (3.4)	2 (2.5)	13 (7.6)	
genotype 3	7 (5.9)	0	4 (2.4)	
genotype other	0	0	0	

Naïve	53 (44.9)	14 (17.5)	43 (25.3)	<0.0001
Experienced	65 (55.1)	66 (82.5)	127 (74.7)	

Relapse	19 (32.2)	35 (56.5)	38 (30.9)	0.009
Partial response	9 (15.3)	7 (11.3)	14 (11.4)	
Breakthrough	5 (8.5)	8 (12.9)	14 (11.4)	
Nonresponse	26 (44.1)	12 (19.4)	57 (56.3)	

HBsAg positive	3 (2.5)	0	1 (0.6)	0.166

HIV positive	0	0	0	N/A

Treatment for hypertension	33 (30.3)	35 (46.7)	72 (47.1)	0.015

Treatment for dyslipidemia	7 (6.4)	11 (14.7)	41 (26.8)	<0.0001

Treatment for T2DM	12 (10.2)	12 (15.0)	34 (20.0)	0.078

Diet only	2 (20)	5 (55.6)	8 (30.8)	0.024
OAD only	5 (50)	4 (44.4)	4 (15.4)	
Insulin	3 (30)	0	14 (53.8)	

Extrahepatic manifestations	23 (19.5)	10 (12.5)	15 (8.8)	0.03

HCC	1 (0.8)	2 (2.5)	5 (2.9)	0.475

**Table 2:** Baseline laboratory parameters and DAA treatment parameters by degree of fibrosis.

	F0-F2 N=118 Absolute (relative) counts or mean ± SEM	F3 N=80 Absolute (relative) counts or mean ± SEM	F4 N=170 Absolute (relative) counts or mean ± SEM	P
HCV RNA IU/mL	2 945 961±640 644	2 428 590±429 297	2 799 155±348 275	0.427 K-W test

Treatment				

3D combo	83 (70.3)	43 (53.8)	96 (57.8)	<0.0001
SOF LDV	21 (17.8)	35 (43.8)	64 (38.6)	
other	14 (11.9)	2 (2.5)	6 (3.6)	

Ribavirin	19 (16.2)	18 (22.8)	106 (63.9)	<0.0001

Treatment duration				

<8 weeks (incomplete)	4 (3.4)	1 (1.3)	4 (2.4)	0.611
8 weeks	2 (1.7)	0	0	
12 weeks	109 (93.2)	76 (95)	155 (93.4)	
24 weeks	2 (1.7)	3 (3.8)	7 (4.2)	

SVR	89 (96.7)	74 (97.4)	144 (96.0)	0.862

Creatinine umol/L (n=366)	139.5±17.5	84.2±6.2	79.9±5.8	<0.0001

CRP mg/dL (n=127)	3.5±0.78	4.3±1.60	1.6±0.19	0.012

Hb g/L (n=368)	142±1.7	144±2	138±1.4	0.023

Neutrophils 10^9^/L (n=258)	4.01±0.18	3.32±0.17	2.8±013	<0.0001

Platelets 10^9^/L (n=368)	206±6.9	200±7.5	132±5	<0.0001

AFP kIU/L (n=240)	5.4±0.9	7.6±0.6	22.3±0.2	<0.0001

Albumin g/L (n=354)	41.6±0.4	41.5±0.4	38.6±0.4	<0.0001

INR (n=335)	1.02±0.02	1.05±0.03	1.13±0.01	<0.0001

AST %ULN (n=281)	133.6±14.5	145.6±10.0	234.3±14.9	<0.0001

ALT %ULN (n=368)	175.40±14.2	166.2±10.8	229.5±13.9	0.002

GMT %ULN (n=281)	154.6±15.6	144.3±14.4	241.5±30.4	0.01

ALP %ULN (n=281)	76.7±4.1	69.1±2.9	92.9±4.1	<0.0001

Bilirubin umol/L (n=368)	12.9±1.0	13.2±0.5	19.7±1.0	<0.0001

Conj. Bil. umol/L (n=184)	5.4±1.6	3.6±0.3	8.5±1.03	0.01

Glucose (mmol/l)	5.75±0.18	5.84±0.17	6.69±0.2	0.001

Total cholesterol (mmol/L) n=328	4.45±0.10	4.69±0.10	4.19±0.08	0.001

HDL-C (mmol/L) n=140	1.34±0.05	1.32±0.07	1.27±0.06	0.622

LDL-C (mmol/L) n=136	2.73±0.12	2.48±0.15	2.33±0.09	0.02

Triglycerides (mmol/L) n=201	1.37±0.08	1.27±0.10	1.54±0.09	0.165

AIP n=140	-0.03±0.03	-0.03±0.05	0.04±0.04	0.272

FORNS	5.74±0.23	6.18±0.23	8.19±0.18	<0.0001

FIB-4	1.8±0.15	2.55±0.27	5.77±0.54	<0.0001

APRI	0.76±0.09	0.9±0.1	2.27±0.18	<0.0001

TELP (kPa)	6.14±0.23	7.61±0.47	20.07±1.27	<0.0001

**Table 3:** Prevalence of impaired fasting glucose and type 2 DM by the degree of fibrosis.

	F0-F2 N=118 Absolute (relative) counts	F3 N=80 Absolute (relative) counts	F4 N=170 Absolute (relative) counts	P
IFG	27 (22.8)	19 (23.8)	46 (27.1)	0.157

T2DM	17 (14.4)	17 (21.3)	54 (31.8)	0.004

IFG or T2DM	44 (37.3)	36 (45.0)	100 (58.8)	0.001

**Table 4:** Baseline levels of fasting glycemia and impaired fasting glucose or type 2 diabetes mellitus prevalence according to treatment experience.

	Naïve Absolute (relative) counts or mean±SEM	Treatment experienced Absolute (relative) counts or mean±SEM	P
All patients			

Glycemia (mmol/L)	5.71±0.15	6.41±0.16	0.006

IFG	19 (17.1)	74 (28.6)	0.008

T2DM	23 (20.7)	65 (25.1)	0.411

IFG or T2DM	42 (37.8)	139 (53.7)	0.005

Only F4 fibrosis patients			

Glycemia (mmol/L)	6.16±0.23	6.86±0.26	0.637

IFG	10 (23.3)	36 (28.3)	0.535

T2DM	13 (30.2)	41 (32.3)	0.921

IFG or T2DM	23 (53.5)	77 (60.6)	0.411

**Table 5:** Association of type 2 diabetes mellitus with various predictors.

	No T2DM (mean±SEM or Absolute (relative) counts	T2DM (mean±SEM or Absolute (relative) counts	P	OR (univariate logistic regression)	95%CI
Age (years)	58±1	63±1	<0.0001	1.04	1.02-1.07

Male sex	115 (42.6)	45 (51.1)	0.162	1.41	0.87-2.28

BMI (kg/m^2^)	26.6±0.27	28.5±0.6	0.001	1.099	1.035-1.167

HCV RNA (IU/mL)	2 776 629±347 176	2 937 405±434 013	0.259	1.225	0.862-1.741

Treatment experienced	187 (69.3)	65 (73.9)	0.411	1.254	0.73-2.16

Elastography (kPa)	12.5±0.86	13.6±1.3	0.5	1.007	0.99-1.03

F4 fibrosis (elastography)	113 (42.2)	54 (61.4)	0.004	2.67	1.45-4.91

F3 fibrosis (elastography)	60 (22.4)	17 (19.3)	0.227	1.58	0.75-3.34

Duration of HCV infection	10.44±0.44	10.56±0.85	0.900	1.002	0.97-1.047

**Table 6:** Predictors of significant (>5%) decrease of glycemia after DAA treatment.

	Glycemia 5% decrease not achieved (mean±SEM or absolute (relative) counts	Glycemia 5% decrease achieved (mean±SEM or absolute (relative) counts	P	OR	95%CI
Age (years)	60±1	61±1	0.303	1.01	0.97-1.04

Male sex	43 (45.3)	25 (30.5)	0.044	0.53	0.26-0.99

BMI (kg/m^2^)	26.9±0.46	27.2±0.44	0.807	1.015	0.93-1.10

HCV RNA (IU/mL)	2 380 969±443 997	3 404 197±595 764	0.092	1.369	0.91-2.06

Treatment experienced	72 (75.8)	66 (80.5)	0.452	1.32	0.64-2.71

Elastography (kPa)	9.6±1.23	11.1±1.41	0.849	1.01	0.98-1.04

F4 fibrosis (elastography)	45 (47.4)	44 (55.0)	0.363	1.43	0.66-3.12

F3 fibrosis (elastography)	28 (29.5)	21 (26.3)	0.829	1.10	0.46-2.62

Thrombocytes x10^9^/L	172±7.7	157±9.5	0.07	0.997	0.994-1.001

APRI	1.43±0.16	1.56±0.16	0.558	1.063	0.87-1.31

Baseline fasting glucose (mmol/L)	5.7±1.8	7.2±2.7	<0.0001	1.431	1.177-1.740

## Data Availability

The data used to support the findings of this study cannot be made available in order to protect patient privacy.
